# Report of a Case of Creutzfeldt-Jakob Disease With an Unusual Quick Evolution

**DOI:** 10.7759/cureus.22982

**Published:** 2022-03-09

**Authors:** Hajar Fadili, Rim Tazi, Hiba El oury, Karim El Aidaoui, Asmaa Hazim

**Affiliations:** 1 Neurology, Cheikh Khalifa Bin Zayed Al Nahyan Hospital, Mohammed VI University of Health Sciences (UM6SS), Casablanca, MAR; 2 Nephrology, Cheikh Khalifa Bin Zayed Al Nahyan Hospital, Mohammed VI University of Health Sciences (UM6SS), Casablanca, MAR; 3 Anesthesia and Critical Care, Cheikh Khalifa Bin Zayed Al Nahyan Hospital, Mohammed VI University of Health Sciences (UM6SS), Casablanca, MAR; 4 Neurology, Mohammed VI University of Health Sciences (UM6SS), Casablanca, MAR

**Keywords:** 14-3-3 protein, neuropathology, rapid evolution, encephalopathy, prion, creutzfeldt-jakob disease

## Abstract

Creutzfeldt-Jakob disease is a rare, transmissible neurodegenerative disorder, most prevalent between the ages of 50 and 70 years old, that is incurable and fatal. It’s caused by a slow, infectious protein agent-designated prion. The most common clinical presentations are sleep disturbances, personality changes, ataxia, aphasia, visual disturbances, weakness, and myoclonus combined with progressive dementia. Here we report the case of a patient with disturbance of consciousness, restlessness, and myoclonia who died two weeks after admission. The analysis of his cerebrospinal fluid reveals that the presence of 14-3-3 protein was positive, which supports the diagnosis of Creutzfeldt-Jakob disease. Our observation underscores the importance of the quick fatality of this case.

## Introduction

Creutzfeldt-Jakob disease (CJD) is a rare neurodegenerative disorder, most prevalent between the ages of 50 and 70 years old, that is incurable and fatal. It's caused by an infectious protein called a prion. It can be hereditary as it can be contagious [1]. This report will discuss the case of a 49-year-old male patient with disturbance of consciousness, restlessness, visual hallucinations, and myoclonus in whom the symptomatology rapidly evolved with a normal brain magnetic resonance imaging (MRI) and died two weeks after admission. This is where the importance of our case lies. Given the normality of brain MRI, the electroencephalogram (EEG) and the study of the patient’s cerebrospinal fluid in search of the 14-3-3 protein are important to guide the diagnosis of Creutzfeldt-Jakob disease.

## Case presentation

This report aims to present the case of a 49-year-old male patient with a Glasgow Coma Scale (GCS) of 12, referred to our institute with no particular medical history, who presented with an encephalopathy associated with reflex myoclonus. The patient was complaining of headaches and visual hallucinations that appeared the day before his admission, in the absence of any infectious or traumatic context. His condition worsened rapidly all at once, and the onset of disturbance of consciousness and myoclonus of the limbs and face prompted emergency consultation and hospitalization. The family interview didn’t find any notion of sting or bite.

**Figure 1:**
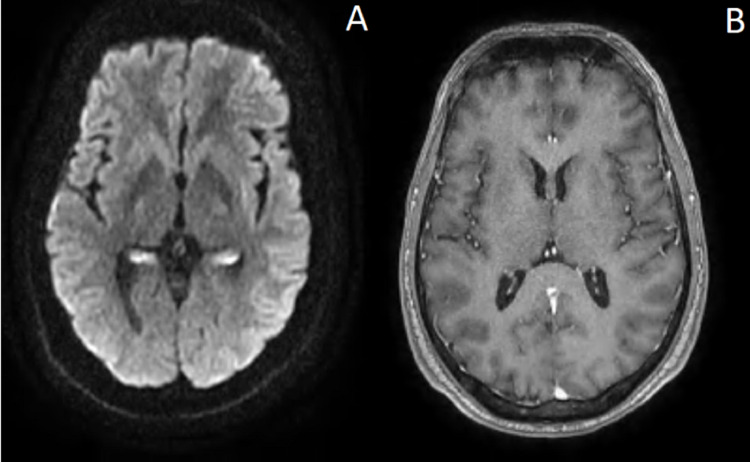
Brain magnetic resonance shows no cerebral abnormality. The cerebral cortex appears to have hyperintensity on diffusion-weighted imaging (DWI). It's an artifact of MRI at our institution. This MRI has been compared to other normal MRI images in our institution and showed no abnormality.

On the other hand, the clinical examination during the admission revealed an afebrile and confused patient in a bad general condition, with no meningeal syndrome, who presents myoclonus at the slightest noise or contact, especially on the face and limbs. As can be seen in Figure 1, the fourth brain MRI showed no cerebral abnormality.

Conversely, the analysis of cerebrospinal fluid was more revealing; it found higher leukocytes at 20/mm^3^ (100% lymphocytes) and increased levels of tau protein. The meningitis and encephalitis panels by polymerase chain reaction (PCR) included *Escherichia coli*, *Haemophilus influenzae*, *Listeria monocytogenes*, *Neisseria meningitidis*, *Streptococcus agalactiae*, *Streptococcus pneumoniae*, cytomegalovirus, tuberculosis, enterovirus, herpes simplex virus (HSV-1, HSV-2), human herpes virus, human parechovirus, varicella zoster virus, and *Cryptococcus neoformans*/*Cryptococcus gattii*. In addition to viral serologies, which include hepatitis, human immunodeficiency virus, syphilis, Lyme disease, and Brucellosis. Moreover, the vitamin dosages (B1, B6, B9, and B12) were normal.

**Figure 2:**
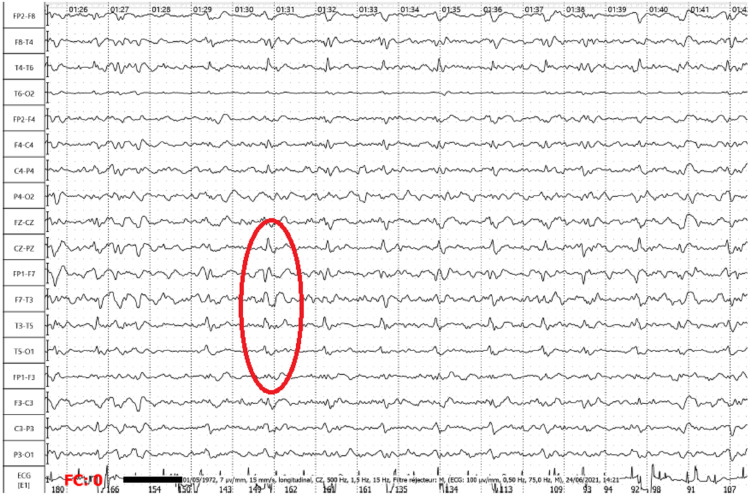
Electroencephalogram shows generalized discharges of short periodic slow-wave spikes at a one-second cycle.

The onconeuronal antibodies (anti-amphiphysine, -CV2, -PNMA2/TA, -Ri, -Yo, -Hu, recoverin, -SOX1, -Titin, -Zic4, anti-GAD65, -Tr (DNER)) were negative. Autoimmune antibodies including (antinuclear antibodies, antineutrophil cytoplasmic antibodies, anticentromere antibodies, antihistone antibodies, cyclic citrullinated peptide antibodies, anti-SS-A, anti-SS-B, anti-RNP, anti-Jo-1, anti-Sm, Scl-70, and rheumatoid factor) were also negative. The search for toxic substances in the blood (methamphetamine, benzodiazepine, barbiturates, paracetamol, cocaine, cannabis, morphine, opioids and lithium, rat poison, cyanide, and arsenic) was negative too. Hence, a 14-3-3 protein search was requested on the cerebrospinal fluid, and it turned out to be positive. Furthermore, the electroencephalogram (EEG) showed disorganization and a diffuse slowing of the background rhythm with generalized biphasic sharp waves, periodic short at one cycle per second, which is pathognomonic of Creutzfeldt-Jakob disease (Figure 2).

**Figure 3:**
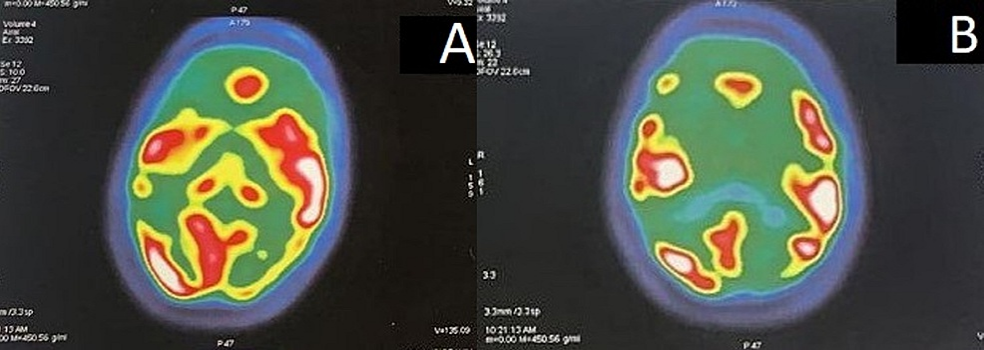
[18F] fluoro-2-deoxy-D-glucose positron emission tomography (FDG-PET); FDG-PET reduced glucose metabolism.

Before receiving the results of the spinal puncture, in search of a neoplastic etiology, our patient received a positron emission tomography (PET) scan, which showed a bilateral symmetric cerebral cortex hypometabolism associated with cerebellar and caudate hypometabolism (Figure 3).

To rule out Hashimoto’s encephalopathy and hepatic encephalopathy, thyroid and liver function tests were requested and turned out to be normal. The dementia was eliminated given the brutality of the installation and the rapid evolution in two weeks. The patient had normal and developed intellectual activity. Two days before the onset of the symptomatology, he could participate in international congresses and give lessons to his students without any difficulty. Knowing that the patient did not have an extrapyramidal syndrome, dementia with Lewy bodies has been eliminated, that's why we were more in favor of encephalopathy. An electroencephalogram (EEG) has been repeated every day and showed the same aspect as the first one. The MRI has been done a second time and stayed normal. After excluding all other potential causes of this symptomatology, given the aspect of the EEG, the positive 14-3-3 protein, and knowing that the definitive diagnosis is based on post-mortem biopsy. Subacute Creutzfeldt-Jakob encephalopathy in its sporadic form remains the most probable premortem diagnosis.

Since his admission, the patient has received hydroelectric supplements, antibiotic therapy, and antiviral treatment (acyclovir), which was stopped after receiving the negative result of herpes simplex virus PCR in the cerebrospinal fluid. Our patient's neurological symptoms evolved quickly over two weeks, and he has been intubated. He caught a nosocomial infection during his hospitalization in the intensive care unit. The starting point was urinary, then he died from a septic shock. The family refused to do the post-mortem biopsy, and the diagnosis of Creutzfeldt-Jakob was retained based on the spinal puncture, the EEG, and the hypometabolism on the PET scan.

## Discussion

Creutzfeldt-Jakob disease is a mortal, contagious neurodegenerative disease caused by an infectious protein agent called a prion [1]. Here, we report the unusual case of a quickly fatal Creutzfeldt-Jacob with a normal cerebral MRI.

The patient presented headaches with disturbances of consciousness, visual hallucinations, and myoclonus of the limbs and face at the slightest noise or contact, especially on the face and limbs. Clinical symptoms are non-specific and may include rapidly progressive dementia, visual disturbances, and various movement disorders like myoclonus, dystonia, tremors, and parkinsonian syndrome. We can find akinetic mutism in the late stages of the disease and eventually death. These findings are challenging to diagnose premortem due to the low clinical suspicion and how rare this disease is [2].

The clinical evolution of our patient was quickly unfavorable compared to other cases in the literature. The disease is fatal in an invariable way, the survival can uncommonly exceed a few months to one year. A study reported that sporadic CJD patients with occipital cortex and basal ganglia hyperintense lesions had a shorter duration of the disease [3]. Our patient had a quick evolution with a normal MRI. This is where the importance of our case lies.

Even though the disease can only be confirmed by the pathologic prion deposition in the brain, demonstrated by a post-mortem biopsy. The diagnosis can be proven by periodic sharp wave complexes on EEG, 14-3-3 protein detection in the cerebrospinal fluid, and abnormal signal changes hyperintensity on both (fluid attenuated inversion recovery, FLAIR) images and diffusion-weighted confined to the gray matter in the cortex, striatum, medial and/or posterior thalamus, or a combination of these areas [4]. It was the same case for our patient, except for the MRI, which showed no abnormalities twice.

In search of a neoplastic etiology, our patient received a positron emission tomography (PET) scan, which showed a bilateral symmetric cerebral cortex hypometabolism associated with cerebellar and caudate hypometabolism. A PET scan uses radioisotopes to detect the level of glucose metabolism in brain cells. The neuronal damage caused by CJD will affect glucose metabolism in brain cells, leading to reduced glucose fixation. But, because PET hypometabolism changes are more common in other neurodegenerative diseases, PET hypometabolism has not been included in the diagnostic criteria for CJD [5].

However, abnormalities on the MRI are not a prerequisite for the diagnosis of Creutzfeldt-Jakob disease. However, diffusion-weighted MRI may show characteristic imaging changes in most patients, independent of the detection of periodic sharp wave complexes on the EEG [6]. Hence the interest of the PET scan, which can show cerebral hypometabolism in the early stages of the evolution well before the appearance of the lesions on the MRI.

Various tests can help orient the clinical diagnosis. The brain biopsy is still the gold standard for diagnosis, but there is a high risk of contamination of the surgical suite and lab. In a study in the National Institute of Neurology and Neurosurgery, in Mexico, even though seven cases died throughout five years, only 43% of them had a confirmed diagnosis, although the clinical profile, the laboratory and imaging studies that oriented the diagnosis in most cases, no post-mortem study could be done because of the refusal of the patients’ families [7] and the same problem was mentioned in our case.

At this moment, no specific treatment exists for CJD. We can only manage the symptoms and the prognosis is very poor [8]. That’s why prevention is the most effective solution. The World Health Organization (WHO) recommended in its guidelines that biological fluids should be handled with care and that effective sterilization and disinfection procedures for instruments should be followed. Also CJD patients, subjects with a family history of the disease, and people who have received biological fluids or implants should be excluded from blood and tissue donation. Sterilizing solutions and specific temperatures for autoclaves used in the sterilization of instruments have also been recommended [7]. Our structure has taken into account the measures as far as possible in order not to contaminate the equipment and to protect the staff and the hospitalized patients. These aseptic measures remain the best prevention of the disease. In addition to that, our hospital declared the disease since it is a notifiable disease.

The limitation of this study was the unavailability of the investigation of 14-3-3 protein in the cerebrospinal fluid and pathogenic variants such as codon 129 in the prion gene. We had to wait for the results to come from abroad before being well oriented.

## Conclusions

We reported the rare case of a quickly fatal Creutzfeldt-Jacob with a normal cerebral MRI. Our observation underscores the importance of quick fatality in this case. The EEG was pathognomonic and 14-3-3 protein was positive, so we must always have the reflex to think of a CJD in front of encephalopathy with myoclonus and search for the protein in the cerebrospinal fluid. It’s necessary to include the post-mortem biopsy as a cornerstone of the approach and to evaluate the results to improve research on this disease, which is still an enigma like the other prion diseases.
